# A comprehensive analysis of immune checkpoint receptor–ligand pairs in aortic diseases highlights the immunosuppressive roles of CD155 and CD274

**DOI:** 10.1016/j.gendis.2025.101724

**Published:** 2025-06-18

**Authors:** Ying Shao, Fatma Saaoud, Keman Xu, Yifan Lu, Sheng Wu, Laisel Martinez, Roberto Vazquez-Padron, Beata Kosmider, Hong Wang, Xiaofeng Yang

**Affiliations:** a Lemole Center for Integrated Lymphatics and Vascular Research, Lewis Katz School of Medicine at Temple University, Philadelphia, PA 19140, USA; b Centers for Metabolic Disease Research and Thrombosis Research, Department of Cardiovascular Sciences, Lewis Katz School of Medicine at Temple University, Philadelphia, PA 19140, USA; c DeWitt Daughtry Family Department of Surgery, Leonard M. Miller School of Medicine, University of Miami, Miami, FL 33136, USA; d Center for Inflammation, Translational and Clinical Lung Research (CILR), Department of Microbiology, Immunology and Inflammation, Lewis Katz School of Medicine at Temple University, Philadelphia, PA 19140, USA

Immune checkpoints (ICPs) are immunologically functional membrane proteins essential for maintaining self-tolerance and regulating immune responses to prevent tissue damage.^1^ These molecules function through receptor–ligand interactions between T cells and antigen-presenting cells, balancing co-stimulatory and co-inhibitory signals. While therapies targeting ICP, such as inhibitors of programmed cell death 1 (PD-1)/programmed cell death ligand 1 (PD-L1) and cluster of differentiation 80 (CD80)/CD86/cytotoxic T-lymphocyte-associated protein 4 (CTLA-4), have transformed cancer treatment, increasing evidence suggests that ICPs are also involved in the pathogenesis of cardiovascular diseases, including abdominal aortic aneurysm (AAA) and atherosclerosis.^2^ Notably, ICP receptors and ligands exhibit context-dependent roles in cardiovascular diseases, with diverse expression patterns and functions.^3^ Building upon our previous findings, identifying T cell-mediated vascular inflammation, including responses from CD4^+^ Foxp3^+^ regulatory T cells (Tregs), plays a critical role in AAA and atherosclerosis, we aimed to comprehensively characterize the ICP landscape in these conditions. To achieve this, we integrated Omics data with quantitative reverse transcription PCR validation to profile 58 receptor–ligand pairs (86 genes) across both murine and human datasets (Table S1). In our analysis of 10 AAA transcriptomic datasets (Fig. 1A), we observed consistent up-regulation of the inhibitory pairs CD47/signal regulatory protein alpha (Sirpa) and selectin P ligand (Selplg)/V-set immunoregulatory receptor (Vsir), as well as the stimulatory pair TNF superfamily member 14 (Tnfsf14)/TNF receptor superfamily member 14 (Tnfrsf14), particularly at early disease stages (Day 7 of angiotensin II infusion) compared with Day 28 in apolipoprotein E deficient (*ApoE*^*−/−*^) aortas. Notably, expression patterns were gender-specific; the CD47/Sirpa pair was up-regulated in females, while only Sirpa was increased in males. Selplg was up-regulated in both sexes, but Vsir remained unchanged, highlighting the complexity of immune modulation in AAA in a gender-dependent manner. These findings suggest that both co-stimulatory and co-inhibitory ICPs are inducible during AAA and may help maintain immune homeostasis and limit disease progression, especially during early stages.

**Figure 1:**
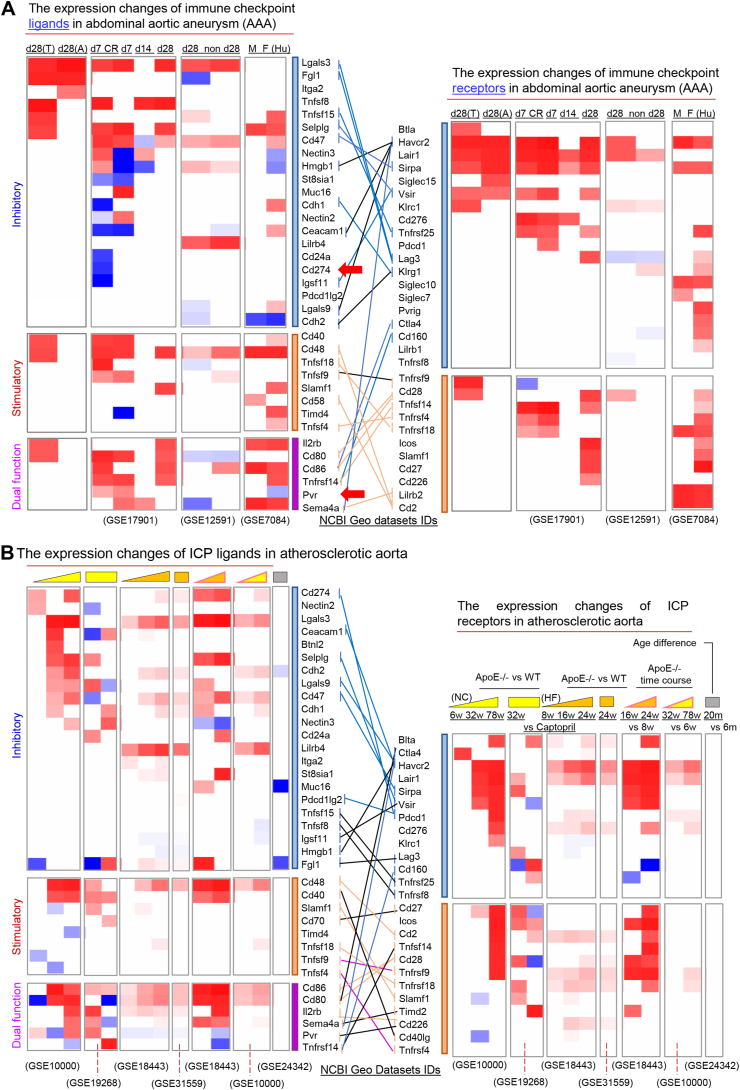
The heatmaps show the differentially expressed immune checkpoint pair (ICP) genes in abdominal aortic aneurysm (AAA) and atherosclerosis development. **(A)** The Log_2_ fold changes (log_2_FC) of differentially expressed genes in collected AAA transcriptomic datasets were visualized in heatmaps. ICP pairs changed in the same dataset are connected by lines. Blue lines indicated up-regulation of inhibitory pairs, orange lines indicated up-regulation of stimulatory pairs, and dark lines showed inconsistent changes between ligands and receptors. **(B)** The log_2_FC of differentially expressed ICP genes in collected transcriptomic datasets of different stages of atherosclerosis were visualized in heatmaps. Additionally, one dataset comparing age difference and one comparing macrophage (Mϕ) versus whole aorta were included as controls. T, thoracic aortic aneurysm (TAA); CR, contained rupture; A, abdominal aortic aneurysm (AAA); d, days after angiotensin II infusion; w, weeks; non, angiotensin II treated mice that did not develop aneurysms; Hu, human; M, male; F, female; NC, normal chow diet; HF, high fat diet; vs, versus; m, months.

To further investigate functional ICP axes, we focused on CD155 (PVR), a multifunctional ligand that interacts with inhibitory receptors, T-cell immunoreceptor with immunoglobulin and immunoreceptor tyrosine-based inhibition motif domain (TIGIT) and CD96, and the stimulatory receptor CD226. For comparison, we included CD274 (PD-L1), a well-characterized inhibitory ligand. CD155 was up-regulated in Day 7 AAA male *ApoE*^*−/−*^ aortas and rupture-prone tissue but down-regulated in female AAA patients, suggesting that its inducibility may resemble that of immunosuppressive cytokines such as interleukin-10 (IL-10) and IL-35, previously reported by our group. In contrast, CD274 was down-regulated on Day 7 AAA tissue prone to rupture. Additionally, Lgals3 was up-regulated in AAA, although its inhibitory receptor lymphocyte activating 3 (Lag3) showed no corresponding change. The inhibitory receptor hepatitis A virus cellular receptor 2 (Havcr2) was significantly induced in AAA, while its semaphorin 4A (Sema4a)-binding competitors, leukocyte immunoglobulin-like receptor B2 (Lilrb2) and T cell immunoglobulin and mucin domain containing 4 (Timd4), were also increased. Interestingly, high mobility group box 1 (Hmgb1), a ligand for Havcr2 in splenocytes and dendritic cells, was increased in groups that failed to develop aneurysms but decreased in AAA mice regardless of sex and female human patients. Furthermore, CD48, which regulates T cell activation via CD2 binding, was significantly up-regulated in both mouse and human AAA. However, CD2 was induced only in human AAA, whereas CD244, another high-affinity ligand for CD48, was increased in mouse AAA. These findings suggest that CD48/CD244 interactions are functionally complex, yielding both stimulatory and inhibitory outcomes.

Our previous studies reported that inhibitory ICP receptors, including TIGIT and PD-1, were up-regulated in Tregs under atherosclerotic conditions, contributing to an immunosuppressive barrier that mitigates disease progression.^4^ To gain a broader understanding of ICP axes during atherosclerosis, we analyzed transcriptomic data from aortas of atherogenic *ApoE*^*−/−*^ and wild-type (WT) mice fed with either a normal diet (ND) or high-fat diet (HFD) at early and late stages of disease. Our results (Fig. 1B) showed prominent induction of co-stimulatory ICP pairs (CD48/CD2, CD86/CD80/CD28) in *ApoE*^*−/−*^ mice compared with WT controls. These expression patterns were further enhanced in late-stage ND-fed *ApoE*^*−/−*^ mice, with HFD accelerating their up-regulation. This suggests a progressive increase in T cell activation as atherosclerosis advances. Interestingly, the inhibitory checkpoint CTLA-4, which competed with CD28 by binding to CD86/CD80 to suppress T cell activation, was only increased in 6 weeks in ND-fed *ApoE*^*−/−*^ mice. In contrast, PD-1 and its ligand CD274 were significantly up-regulated at 78 weeks in HFD-fed *ApoE*^*−/−*^ mice. These observations are consistent with prior studies showing that CTLA-4 exerts regulatory functions in early-stage atherogenesis, while PD-1 acts during later stages.^5^ Furthermore, ligand expression for CD48 and CD80/CD86 increased over time, whereas receptor expression remained unchanged, suggesting early antigen-presenting cell-driven T cell activation or possible saturation-like receptor dynamics.

We also observed the induction of inhibitory pairs Selplg/Vsir and CD47/Sirpa in ND-fed *ApoE*^*−/−*^ mice at 32 and 78 weeks, compared with both WT controls and younger *ApoE*^*−/−*^ mice at 6 weeks. To determine whether hypercholesterolemia and atherosclerotic lesions, rather than age differences, contributed to the inductions, we examined a dataset comparing 6- and 20-month-old WT mice and found no significant changes in ICP expression. Except for a slight increase in CD47, the inductions of *Selplg/Vsir* and *Sirpa* were not observed in HFD-fed *ApoE*^*−/−*^ mice before 24 weeks. Additionally, Havcr2 and Lgals3 were induced with disease progression, although expression of their corresponding ligand for Havcr2 or receptor for Lgals3 remained unchanged*.* CD155 expression increased in ND-fed *ApoE*^*−/−*^ mice but slightly decreased in those on an HFD, with no significant changes observed in its receptor in these datasets, which are likely due to the low frequency of Tregs in atherosclerotic aortas.

To validate our transcriptomic finding, and in accordance with our IACUC-approved protocol at Temple University, we performed quantitative reverse transcription PCR on aortas collected from 12-week HFD-fed *ApoE*^*−/−*^ mice and WT controls, the same mouse model from our previous Treg RNA sequencing study^4^ (Fig. S1). Consistent with our data-mining analysis, CD155 and CD274 expression levels were significantly reduced in *ApoE*^*−/−*^ aortas, while CD86 levels were increased, supporting the immunosuppressive roles of CD155 and CD274 during atherosclerosis. As expected, TIGIT expression was undetectable at this stage, likely due to the limited Treg presence in the aorta.

While quantitative reverse transcription PCR provides high sensitivity and specificity for gene transcript-level quantification, it does not fully reflect protein abundance. Therefore, future studies should incorporate protein-level validation, such as western blotting, flow cytometry, or immunohistochemistry, to confirm these transcriptomic findings and fully elucidate ICP function in aortic disease. We also acknowledge the discrepancy between transcriptomic and quantitative reverse transcription PCR findings for TIGIT and highlight possible contributing factors such as tissue heterogeneity, low cellular abundance, and regulatory complexity.

In summary, this study provides a comprehensive transcriptomic and quantitative reverse transcription PCR-based analysis of 58 immune checkpoint receptor–ligand pairs (86 genes) in AAA and atherosclerosis. We reveal disease stage-, gender-, and hyperlipidemia/normolipidemia-specific expression patterns of both co-stimulatory and co-inhibitory ICPs. Notably, CD155 and CD274 exhibited dynamic and context-dependent regulation, suggesting significant roles in immune modulation throughout disease progression. We also identify key regulatory axes, such as CD47/Sirpa and Selplg/Vsir in AAA, and CD48/CD2, CD86/CD80, and CTLA-4/PD-1 in atherosclerosis, as potential therapeutic targets. These insights highlight the translational potential of targeting immune checkpoints to modulate cardiovascular inflammation and aortic disease.

## CRediT authorship contribution statement

**Ying Shao:** Writing – review & editing, Writing – original draft, Validation, Software, Methodology, Formal analysis, Data curation, Conceptualization. **Fatma Saaoud:** Writing – review & editing, Writing – original draft, Software, Methodology, Investigation, Formal analysis, Data curation, Conceptualization. **Keman Xu:** Writing – review & editing, Formal analysis, Conceptualization. **Yifan Lu:** Writing – review & editing, Formal analysis, Conceptualization. **Sheng Wu:** Writing – review & editing, Conceptualization. **Laisel Martinez:** Writing – review & editing, Conceptualization. **Roberto Vazquez-Padron:** Writing – review & editing, Formal analysis, Conceptualization. **Beata Kosmider:** Writing – review & editing, Conceptualization. **Hong Wang:** Writing – review & editing, Supervision, Project administration, Funding acquisition, Formal analysis, Conceptualization. **Xiaofeng Yang:** Writing – review & editing, Writing – original draft, Visualization, Supervision, Resources, Project administration, Funding acquisition, Formal analysis, Data curation, Conceptualization.

## Funding

This work was partially supported by NIH grants to XY (HL163570-01A1 and HL147565-01), HW (DK113775) and NIH T32 training grants to YL (5T32HL091804-15).

## Conflict of interests

The authors declared no conflict of interests.
