# Supplementary material for: A comprehensive analysis of immune checkpoint receptor–ligand pairs in aortic diseases highlights the immunosuppressive roles of CD155 and CD274
Source: Genes Dis. 2025 Jun 18;13(2):101724. doi: 10.1016/j.gendis.2025.101724 (PMC12596592; doi:10.1016/j.gendis.2025.101724)
Supplement: Multimedia component 1 [file mmc1.docx]

**Supplementary data**

**Methods and materials**

*Transcriptomic analyses of immune checkpoints in the aorta from abdominal aortic aneurysm (AAA), and hyperlipidemia and atherosclerosis mouse models*

Expression profiles of immune checkpoint genes in the aorta were collected from 10 published studies deposited in the NIH-NCBI Geo DataSets database (<https://www.ncbi.nlm.nih.gov/gds/>), as we previously reported. The datasets IDs were included in Figure 1, along with one set of our own RNA-sequencing results. These datasets included three mouse and one human AAA models, and six hyperlipidemia and atherosclerosis models. To avoid bias resulting from differences between each independent experimental design and technology, we used the log fold changes (logFC) in each specific experimental comparison to generate a heat map.

**Supplementary Table 1. The expressions of 58 immune checkpoint pairs (86 genes) were examined, including 1 dual function pair, 36 inhibitory pairs, and 21 stimulatory pairs.** As the arrows indicated, CD155 (poliovirus receptor, PVR) is a ligand in 3 ICPs including two inhibitory pairs (CD155-TIGIT and CD155-CD96), and one stimulatory pair (CD155-CD226). In comparison, CD274 is a ligand with single partner in one inhibitory pair (CD274-PD-1 (PDCD1, CD279). Of note, some ligands share receptors, and some receptors share ligands, so that the total number of genes is 86, not 116, after removing duplicates and unidentified genes.

| **Category** | **APC** | **T cell** | **PMID** |
| --- | --- | --- | --- |
| Both | TNFRSF14 | BTLA | 15568026, 15647361 |
| Inhibitory | BTNL2 | UNKNOW | 16751379 |
|  | CD24A | SIGLEC10 | 19264983 |
|  | CD274 | PDCD1 | 11015443 |
|  | SIRPA | CD47 | 9872987 |
|  | CD80 | CTLA4 | 7543139, 7545666 |
|  | CD86 | CTLA4 |  |
|  | CDH1 | KLRG1 | 19654330 |
|  | CDH2 | KLRG1 |  |
|  | CEACAM1 | HAVCR2 | 25363763 |
|  | FGL1 | LAG3 | 30580966 |
|  | HHLA2 | KIR3DL3 | 34244312 |
|  | HLA-G | KIR2DL4 | 18334671 |
|  | HMGB1 | HAVCR2 | 22842346 |
|  | IGSF11 | VSIR | 30220083 |
|  | CD29 | CD49B | 7523399, 23624599 |
|  | LGALS3 | LAG3 | 25691328 |
|  | LGALS9 | HAVCR2 | 11823861 |
|  | LILRB4 | UNKNOW | 9151699 |
|  | MHC-I | LILRB1 | 29180808 |
|  | MUC16 | SIGLEC9 | 20497550 |
|  | GD3 | SIGLEC7 | 12778482 |
|  | NECTIN2 | TIGIT | 19011627 |
|  | NECTIN2 | PVRIG | 26755705 |
|  | NECTIN3 | TIGIT | 19011627 |
|  | PDCD1LG2 | PDCD1 | 11224527 |
|  | PVR | TIGIT | 19011627 |
|  | PVR | CD96 | 15034010 |
|  | VSIR | SELPLG | 31645726 |
|  | TNFRSF14 | CD160 | 18193050 |
|  | TNFSF15 | TNFRSF25 | 24242819 |
|  | TNFSF8 | TNFRSF8 | 19760074 |
|  | UNKNOW | SIGLEC15 | 30833750 |
|  | HLA-E | KLRC1 | 37675109 |
|  | UNKNOW | LAIR1 | 9285412 |
|  | UNKNOWN | VTCN1 | 12818165 |
|  | NECTIN1 | CD96 | 30759143 |
| Stimulatory | CD47 | SIRPG | 34925315 |
|  | UNKNOW | CD276 | 11224528 |
|  | CD40 | CD40LG | 1282319 |
|  | CD48 | CD2 | 7691954 |
|  | CD58 | CD2 | 8757306 |
|  | CD70 | CD27 | 7547681 |
|  | CD80 | CD28 | 7545666 |
|  | CD86 | CD28 |  |
|  | HHLA2 | TMIGD2 | 25549724, 23784006 |
|  | ICOSLG | ICOS | 9930702, 11983910 |
|  | PVR | CD226 | 12913096 |
|  | SEMA4A | LILRB2 | 29467366 |
|  | SEMA4A | TIMD2 | 12374982 |
|  | SLAMF1 | SLAMF1 | 9126961 |
|  | TIMD4 | HAVCR1 | 15793576 |
|  | TNFSF14 | TNFRSF14 | 8898196 |
|  | TNFSF18 | TNFRSF18 | 10074428 |
|  | TNFSF4 | TNFRSF4 | 7749983 |
|  | TNFSF9 | TNFRSF9 | 8405064 |
|  | NCR3 | NCR3LG1 | 19528259 |
|  | CD48 | CD244 | 9841922 |

*RNA-seq*

0.66

0.81 (LogFC value)

0.36

-0.37

0.98

0.41

0.71

-0.5

*

*

**A**

*

**B**

**Supplementary Figure 1. The expression levels of CD155 and CD274 were significantly decreased in the ApoE-/- aorta compared to those of WT. The 12-week (w)-high fat diet (HFD)-fed ApoE^-/-^ and WT mice (n > 8 per group) were used for analysis. A) Differential expressions of 8 ICP receptors were found in spleen CD4^+^Foxp3^+^ Tregs from 12-wk HFD-fed ApoE^-/-^ (n=4) and WT male mice (n=3), with log2FC indicated above columns (p-value < 0.05). B) The expressions of ICP ligands CD86, CD274 (PD-L1), and PVR (CD155) in the aorta were examined by real-time PCR.**
